# Highly Sensitive Levodopa Determination by Means of Adsorptive Stripping Voltammetry on Ruthenium Dioxide-Carbon Black-Nafion Modified Glassy Carbon Electrode

**DOI:** 10.3390/s21010060

**Published:** 2020-12-24

**Authors:** Anna Górska, Beata Paczosa-Bator, Robert Piech

**Affiliations:** Faculty of Materials Science and Ceramics, AGH University of Science and Technology, al. Mickiewicza 30, 30-059 Kraków, Poland; agorska@agh.edu.pl (A.G.); paczosa@agh.edu.pl (B.P.-B.)

**Keywords:** levodopa, voltammetry, ruthenium dioxide nanoparticles, Nafion, carbon black

## Abstract

A new, highly sensitive Adsorptive Stripping Voltammetric method for levodopa determination was developed. As a working electrode, the glassy carbon electrode (GCE) modified with carbon black (CB), RuO_2_·xH_2_O (RuO_2_) and Nafion was used (CB-RuO_2_-Nafion GCE). Levodopa signal obtained on the modified electrode was 12 times higher compared to GCE. During research, instrumental parameters were optimized: sampling time t_s_ = 10 ms, waiting time t_w_ = 10 ms, step potential E_s_ = 5 mV and pulse amplitude ΔE = 50 mV. Preconcentration potential E_prec_ was equal to 0 mV. The best results were obtained in 0.025 M perchloric acid (approx. pH 1.4). Signal repeatability measured on the CB-RuO_2_-Nafion modified electrode for 0.2 µM of levodopa was equal to 2.1% (levodopa concentration 1 µM, *n* = 5). Linearity of the method was achieved in the concentration range from 1 to 8 µM. Limit of detection was equal to 17 nM. Recoveries calculated for pharmaceutical products and tap water measurements were in the range 102–105%, which confirms the accuracy of the developed. The applicability of the method was confirmed by analysis of pharmaceutical products and tap water samples. Based on obtained results, it might be concluded that the developed voltammetric method could be a useful tool in routine drug analysis.

## 1. Introduction

Levodopa (dihydroxyphenylalanine, DOPA) is a large neutral amino acid (LNAA) used in therapy of Parkinson’s disease. Literature states that to date levodopa has the best therapeutic index from symptomatic parkinsonian medications [1,2]. Considering the role of levodopa in Parkinson’s disease treatment, it might be concluded that a quality control on medications containing this substance is an important issue. Literature describes application of different instrumental methods for levodopa determination. Among them is high-pressure liquid chromatography (HPLC) with diod-array detection (DAD) [3], UV [4], fluorimetric [4] or chemiluminescence [5] detection. Another method in capillary electrophoresis with chemiluminescence detection [6]. The use of fluorescence spectrometry [7] and spectrophotometry [8] was also reported. Voltammetry which belongs to the group of electrochemical methods is also a useful tool for Levodopa determination [9,10,11,12,13,14,15,16,17]. In comparison to the mentioned methods, voltammetry has many advantages. It is relatively inexpensive, samples analysis is easy and fast and most importantly, it is a highly sensitive method which allows very low detection limits to be reached.

Voltammetry might be used for determination of organic and inorganic substances. This is possible due to numerous design solutions in the field of working electrodes [18,19,20,21,22,23,24]. A very popular type of sensor is the glassy carbon electrode (GCE) due to the possibility of its modification with surface modifier. Application of such materials allows parameters such as sensitivity, selectivity or limit of detection to be improved. In the literature examples of application of glassy carbon electrodes [25,26,27,28] and carbon paste electrodes [29] modified with ruthenium in the form of oxides, hydrated oxides or organic compounds might be found. Ruthenium exhibits unique properties such as high conductivities, unusual stability and easy electron transfer. These properties make ruthenium a great electrocatalyst which increases the current response when it is used as electrode material in electrochemical analysis [28,29,30,31]. In order to improve analytical performance of ruthenium-based sensors, researchers started to combine it with carbon nanomaterials [30,31,32]. Carbon black, graphene, carbon nanotubes and other carbonaceous materials are well known as GCE and CPE modifiers. They might be characterized by high electrical conductivity which improves the performance of a sensor. Therefore, their combination with ruthenium allows the electrode material with interesting properties to be obtained.

The aim of this work was to develop a highly sensitive and simple voltammetric method for levodopa determination. The main novelty of this work was the preparation of the sensor modified with combination of carbon black, RuO_2_·xH_2_O and Nafion and its application for levodopa determination. As it was explained, ruthenium and carbon nanomaterials exhibit properties desired in electrochemical sensors; therefore, they were used as surface modifiers. Nafion was added in order to improve mechanical properties. The abovementioned ruthenium-based sensor is easy to prepare and might be characterized high sensitivity, which was shown in our previous work [33]. To the best of our knowledge, this solution has never been used in levodopa determination.

## 2. Experiment

### 2.1. Apparatus

Voltammetric experiments were conducted using an electrochemical analyzer M161 (mtm-anko) and electrode stand M164 (mtm-anko). A typical three-electrode system, consisted of RuO_2_-CB-Nafion GCE (working electrode), Ag/AgCl (3M KCl) (reference electrode) and platinum wire (auxiliary electrode), was applied. The solution in the quartz cell (total volume 20 mL) was stirred using magnetic bar rotating with the speed ~500 rpm. All pH measurements were carried out with a laboratory pH-meter (N-512 elpo, Polymetron, Wroclaw, Poland). For solvents, a sonication ultrasonic bath (BAKU BK-3550 30/50W, frequency 40 kHz) was used.

### 2.2. Chemical and Glassware

All reagents were of analytical grade. The stock solution of levodopa was prepared from certified reference material (Sigma Aldrich, St. Louis, MO, USA). In order to stabilize the levodopa solutions, perchloric acid (Sigma Aldrich, Darmstadt, Germany was added (10 µL of acid per 1 mL of solution). The stock solution of levodopa was stored in the fridge at a temperature of 4 °C. Solutions with lower concentration were prepared daily by dilution of the stock solution. The remaining reagents were purchased as follows: Carbon black CAT (surface area 550 m^2^ kg^−1^)–3D Nano; RuO_2_·xH_2_O–Alfa Aesar, A Johnson Matthey Company; ethanol–POCH, Gliwice Poland; Humic acid sodium salt–Aldrich; Triton X-100—Sigma Aldrich, Darmstadt, Germany, Nafion–Sigma Aldrich, Darmstadt, Germany Prior to use, all glassware was cleaned using HNO_3_ solution, then rinsed with double distilled water. Double distilled water was used in all measurements.

### 2.3. Sample Preparation

For the study of levodopa concentration in pharmaceutical products, Madopar 125–100 mg per capsule and Madopar 62.5–50 mg per tablet were used. In the first step of sample preparation, 3 tablets from each pharmaceutical product were weighed, crushed using the agate mortar and mixed. In the next step, the adequate amount of each sample was dissolved in double distilled water (20 mL) and then sonicated for about 10 min. In order to remove the tablet’s insoluble fillers, each sample was filtrated and obtained filtrate was transferred into the 25 mL volumetric flask. Samples were stabilized by addition of 250 µL of perchloric acid and filled up to the mark with double distilled water.

### 2.4. The Preparation of Working Electrode

**Figure 1:**
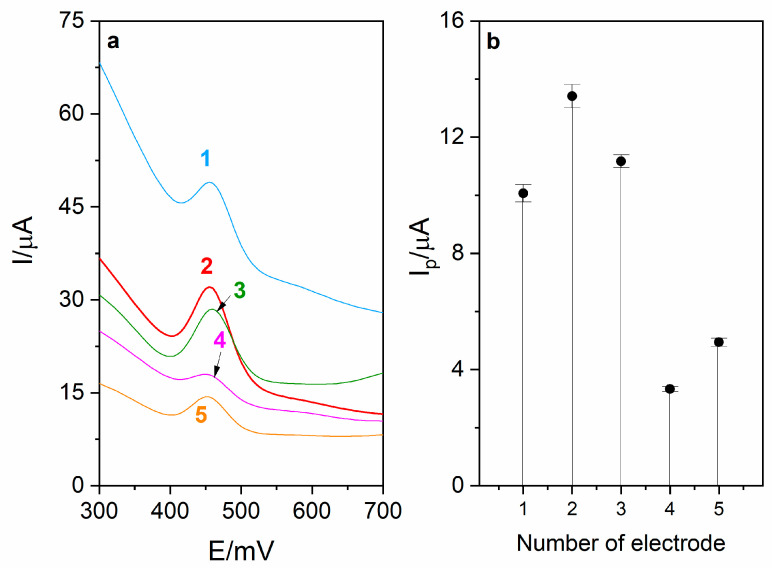
(**a**) Voltammograms obtained during optimization of modifier composition. Each modifier was prepared in 5 mL volumetric flask using the following amounts of carbon black (CB) and RuO_2_·xH_2_O (RuO_2_): (1)—10 mg CB, 1 mg RuO_2_, (2)—5 mg CB, 1 mg RuO_2_, (3)—5 mg CB, 2 mg RuO_2_, (4)—2.5 mg CB, 1 mg RuO_2_, (5)—5 mg CB, 0.5 mg RuO_2_. (**b**) Corresponding diagram.

The first step of RuO_2_-CB-Nafion GCE fabrication is preparation of CB–RuO_2_–Nafion dispersion (modifier). For this purpose, 5 mg of carbon black and 1 mg of RuO_2_·xH_2_O were weighed and quantitatively transferred into a 5 mL volumetric flask. In the next step, 100 μL of Nafion (5%) was added, and then the flask was filled up to the mark using ethanol (96%). The mixture was sonicated for about 15 min. Amounts of substrates listed above were optimized. In Figure 1, voltammograms obtained during the optimization of carbon black and RuO_2_·xH_2_O are presented as well as the corresponding diagram. During the experiment, different ratios between mentioned components were tested and the best combination was chosen for preparation of surface modifier. 

The second step of RuO_2_-CB-Nafion GCE fabrication is modification of the GCE using obtained modifier. Firstly, the surface of the GC electrode has to be cleaned in order to remove adsorbed contamination. For this purpose, the electrode is polished using alumina powder suspension with particle sizes of 0.3 and 0.05 µm and polishing cloth. Then, the electrode is rinsed in the double distilled water stream, put in the methanol-water solution and sonicated for about 3 min. After drying, the surface of the GC electrode is modified by application of 5 µL of modifier suspension (homogenized directly before use by 3 min sonication). After drying in the room temperature, which takes at least 15 min, the sensor is ready to use and might be utilized for a few weeks. The results of the experiment concerning stability of RuO_2_-CB-Nafion GCE can be found in our previous work [33]. The sensor reproducibility was also tested. A series of RuO_2_-CB-Nafion GCE sensors was prepared, and then each of them was tested in the same conditions. Results shown that reproducibility (expressed as variation coefficient) of the sensor is equal to 7.3% (*n* = 5). After use between different measuring days, the sensor was rinsed using double distilled water and stored at room temperature in the glass container to avoid contamination from the air. 

### 2.5. Measurement Procedure

Quantitative measurements of levodopa were carried out using differential pulse voltammetry (DPV) and the standard addition procedure. Measurements were carried out in the supporting electrolyte which consisted of 0.025 M perchloric acid (pH 1.4)—total volume 10 mL in the voltammetric cell. Before the first use of the electrode, its signal was stabilized by cycling (to achieve a stable signal). The measurement procedure was performed following the steps below:

- Cleaning of the electrode surface: E = 1205 mV, t = 2 s;
- Preconcentration step: E_acc_ = 0 mV, t_acc_ = 20 s;
- Rest period: 3 s;
- Registration of voltammogram in the potential range from 0 to 1200 mV.

Other parameters of DPV measurements were as follows: waiting time t_w_ = 10 ms; sampling time t_s_ = 10 ms; step potential E_s_ = 4 mV; and pulse amplitude ΔE = 50 mV.

## 3. Results and Discussion

### 3.1. Voltammetric Behavior of Levodopa on CB-RuO_2_-Nafion GCE

**Figure 2:**
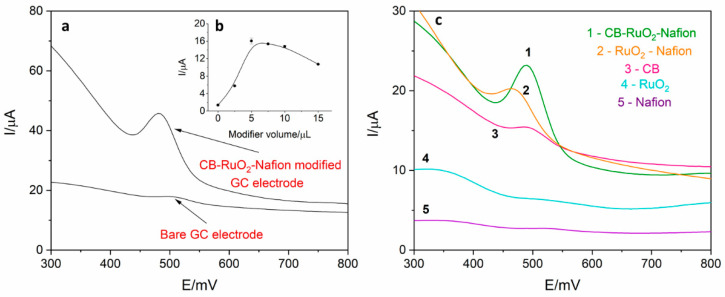
(**a**) Comparison of differential pulse voltammetry (DPV) signal derived from 2 µM of levodopa register on RuO_2_-CB-Nafion glassy carbon electrode (GCE) and on bare GCE electrode in 0.025 M perchloric acid. Preconcentration potential and time were equal to 0 mV and 20 s, respectively. (**b**) Dependence of levodopa peak current on modifier volume. (**c**) Comparison of signals obtained using glassy carbon electrode modified with: 1—carbon black, RuO_2_ and Nafion, 2–RuO_2_ and Nafion, 3—carbon black, 4—RuO_2_, 5—Nafion. Levodopa concentration was equal to 4 µM; supporting electrolyte consisted of 0.025 M perchloric acid; preconcentration potential and time were equal to 0 mV and 20 s, respectively.

**Figure 3:**
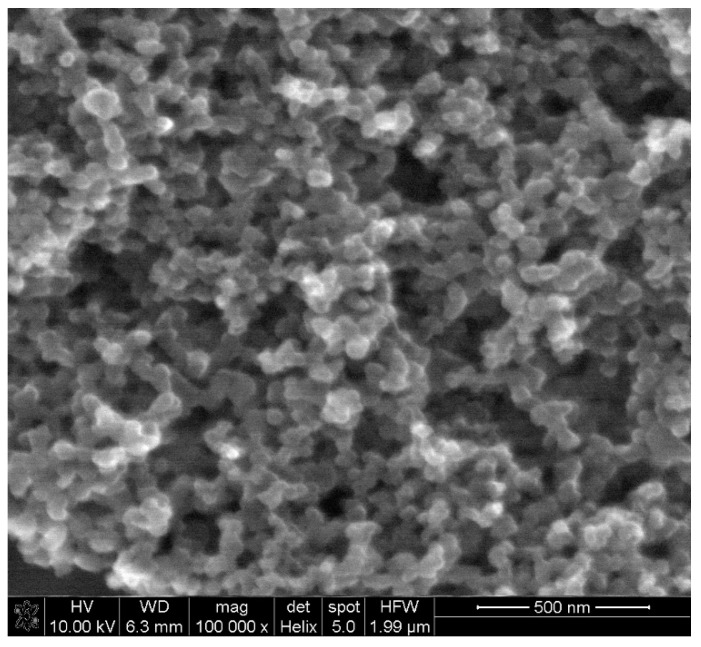
SEM image of RuO_2_-CB-Nafion GCE.

Electrochemical behavior of levodopa on the RuO_2_-CB-Nafion GCE and bare GC electrode was compared and is presented in Figure 2a. The test was performed using the surface modifier with optimized composition, which is described in point 2.4. In Figure 3, the characterization of morphology of RuO_2_-CB-Nafion GCE performed by scanning electron microscopy is presented. Carbon black might be characterized by the spherical shape with an average particle size equal to 30 nM. The second component of the electrode modifier–hydrated ruthenium dioxide has a diameter of 2 nM. An initial observation might be the difference in sensitivity between the bare and modified electrode. Peak current registered on RuO_2_-CB-Nafion GCE and the bare electrode was equal to 1.4 and 16.5 µA, respectively. This means that modifications enabled an increase in registered current almost by 12 times. A shift in a peak potential to lower potentials values (bare GCE 510 mV, RuO_2_-CB-Nafion GCE 484 mV) is also visible. This may indicate that the modified electrode has good catalytic properties. The levodopa half width peak for both electrodes are comparable. In Figure 2c, the comparison of levodopa signal measured using glassy carbon electrodes modified with each component of the final modifier (CB-RuO_2_-Nafion) is presented. As it can be seen, when components were used separately, a moderate effect was observed—only their combination resulted in a significant improvement of the sensor’s sensitivity. 

The influence of modifier volume on levodopa peak current was also investigated, which is presented in Figure 1b. Increasing the modifier volume caused an increase in recorded currents (from 1.4 to 16.5 µA) but only up to the volume of 5 µL. For volume larger than 5 µL, the drop in the peak current and increase in the background current were observed, which was probably the effect of increasing capacitive current. Therefore, in further measurements, the GC electrode modified with 5 µL of surface modifier was used. For optimized RuO_2_-CB-Nafion GCE parameters, repeatability was calculated and was equal to 2.1% (levodopa concentration 1 µM, *n* = 5). 

**Figure 4:**
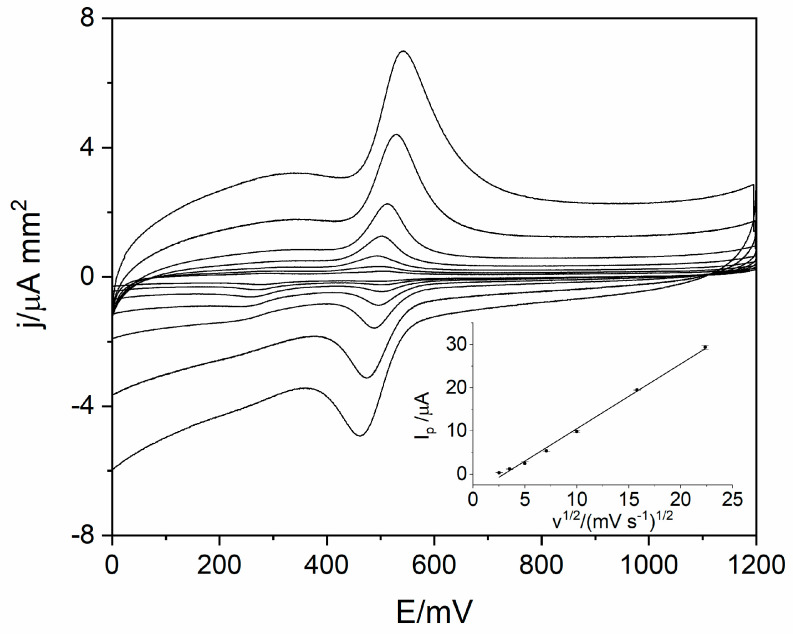
Voltammograms obtained using the linear sweep voltammetry (LSV) technique for scan rate in the range from 6.3 to 500 mVs^−1^ for 10 µM of levodopa in 0.025 M perchloric acid. The dependence of levodopa peak current on square root of scan rate (inset).

In order to identify the reaction mechanism of levodopa on RuO_2_-CB-Nafion GCE, linear sweep voltammetry (LSV) measurements were conducted. Voltammograms for 10 µM of levodopa were registered at different scan rates (6.3–500 mV s^−1^) in the potential range from 0 to 1200 mV, which is presented in Figure 4. The results showed the quasi-reversible nature of the electrode process. The oxidation peak occurs at the potential 512 mV (scan rate 100 mVs^−1^) and corresponds to the oxidation of levodopa to dopaquinone. The reduction peak which occurs at the potential 488 mV (scan rate 100 mVs^−1^) corresponds to the reverse reaction (reduction of dopaquinone to levodopa) [12,17]. The influence of scan rate on LSV voltammograms is also visible in Figure 4—the higher the value of scan rate was, the higher levodopa peak current was. The dependence of peak current versus scan rate and square root of the scan rate were plotted. Obtained diagrams revealed the linear dependence between levodopa peak current and square root of the scan rate (Equation (1)), which means that the electrode process is diffusion controlled (Figure 4 inset).

I_p_ = 1.50 (mV s^−1^)^1/2^ − 4.45 µA r = 0.996(1)

To determine the number of electrons taking part in the electrode reaction, the dependence between peak potential and natural logarithm of the scan rate was plotted (Equation (2)). In the next step, the slope of the regression line was used in the Equation (3). Calculations revealed that the αn value was equal to 1.85, which indicates that the number of electrons (*n*) which take part in the electrode process is equal to 2.

E_p_ = 0.014 ln (V s^−1^) + 0.547 V r = 0.988(2)

b = RT/2αnF(3)

b—slope [-], R—gas constant [J·M^−1^K^−1^], T—temperature [K], α—transfer coefficient [-], n—stoichiometric number of electrons involved in an electrode reaction [-], F—Faraday constant [C·mol^−1^]

**Figure 5:**
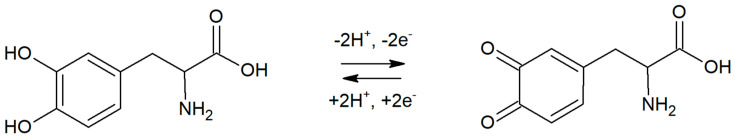
The proposed mechanism of the redox reaction of levodopa on the surface of RuO_2_-CB-Nafion GCE.

In order to confirm the mechanism of the reaction, the influence of the pH of supporting electrolyte on peak potential was studied. In the experiment, six electrolytes with pH in the range from 5.7 to 8 were prepared and tested. Levodopa concentration was equal to 2 µM, and preconcentration potential and time were 0 mV and 20 s, respectively. In a studied range of pH, a linear dependence between pH and levodopa peak potential was obtained. The slope of the regression was equal to 59.7 mV pH^−1^, which is close to the theoretical value (59.17 mV pH^−1^). This indicates that 1 proton per 1 electron takes part in the levodopa electrode reaction. A probable levodopa redox reaction has been already proposed by other researchers [12,13,15,17]. Data presented in this work confirm previous results; therefore, a possible levodopa oxidation mechanism on RuO_2_-CB-Nafion GCE was presented in Figure 5. 

### 3.2. Influence of DPV Technique Parameters on Levodopa Signal

DPV technique parameters influence the sensitivity of the method; therefore, their optimization is an important step of the research. The following parameters were investigated in the wide range of values: sampling time t_s_ (10–80 ms), waiting time t_w_ (10–80 ms), step potential E_s_ (1–6 mV), pulse amplitude ΔE (5–100 mV–positive and negative mode). The best results were obtained for values t_s_ = 10 ms, t_w_ = 10 ms, E_s_ = 5 mV, ΔE = 50 mV; therefore, such parameters were used in further measurements. 

### 3.3. Influence of Preconcentration Potential and Time on Levodopa Signal

Preconcentration potential and time are the parameters of voltammetric technique–adsorptive stripping voltammetry. This technique allows the improvement of the sensitivity of the method by introducing the preconcentration step during which the analyte accumulates on the surface of the working electrode. Therefore, optimization of mention parameters was the next step of the research. The experiment was conducted in 0.025 M perchloric acid, for levodopa concentration equal to 2 µM. Preconcentration potential was varied from −400 to 425 mV. In the range from −400 to 200 mV, register levodopa current practically does not depend on preconcentration potential (the peak current was approximately 16.3 µA). For potentials higher than 200 mV, a decrease in the levodopa peak current was observed. Considering the obtained results, the preconcentration potential equal to 0 mV was chosen as optimal for further measurements. 

The preconcentration time was examined in the same conditions as preconcentration potential for the same levodopa concentration. The time was varied in the range from 0 to 60 s. Increasing the preconcentration time resulted in an increase in the levodopa peak current, but an effective peak current increase for a preconcentration time no longer than 20 s was observed. For preconcentration time longer than 20 s, practically no increase in analytical signal was observed. Preconcentration time equal to 20 s was chosen as optimal due to relatively high sensitivity and ability to perform quick measurement.

### 3.4. Influence of the Composition of Supporting Electrolyte on Levodopa Signal

#### 3.4.1. Composition of Supporting Electrolyte

The type of supporting electrolyte may affect levodopa peak current and potential. Seven different electrolytes in the pH spectrum from 1.4 to 9.1 were chosen for the experiment: borate buffer (0.025 M, pH 9.1), ammonium buffer (0.025 M, pH 8.2), acetate buffer (0.025 M, pH 3.8), KCl (0.025 M, pH 4.8), KH_2_PO_4_ (0.025 M, pH 4.6), H_2_SO_4_ (0.025 M, pH 1.6) and HClO_4_ (0.025 M, pH 1.6). The signal derived from 2 µM of levodopa was obtained in ammonium buffer, KCl, KH_2_PO_4_, H_2_SO_4_ and HClO_4_. Nevertheless, measurement conducted in acids (H_2_SO_4_ and HClO_4_) might be characterized by the highest sensitivity. Comparison of peak current for both acids revealed that signal registered in HClO_4_ (peak current and potential equal to 16.5 µA and 488 mV, respectively) was 35% higher than in H_2_SO_4_. Therefore, perchloric acid was chosen as optimal for voltammetric levodopa determination. 

#### 3.4.2. Influence of Concentration of Perchloric Acid on Levodopa Signal

Further research was focused on the influence of perchloric acid concentration on levodopa signal. Experiment was conducted using perchloric acid with concentration in the range from 0.025 to 0.5 M (levodopa concentration in the system was equal to 2 µM). The higher the electrolyte concentration was, the lower the observed peak current was. In addition, for lower concentration of the electrolyte, a shift in peak potential toward lower values was observed. Further experiments were conducted using 0.025 M perchloric acid, and this value was chosen as optimal. Supporting electrolyte should be characterized by good conductivity; therefore, concentrations lower than 0.025 M were not tested in the experiment. Additionally, lower concentration of supporting electrolyte would generate higher background current.

### 3.5. Intereferences

Considering possible application of the developed method in real samples analysis (pharmaceuticals products and natural water samples), an interferences study was conducted. For the experiment, inorganic cations and anions as well as organic substances were chosen. All used substances with the information about their concentration in the supporting electrolyte are presented in Table 1. During the experiment, the concentration of levodopa was equal to 1 µM. The results show that the presence of the higher concentration of Fe(III) (more than 0.5 µM) caused a decrease in the signal (1 µM of Fe(III) generated 15% decrease in the peak current). Other inorganic ions did not affect register current. From the organic substances, Triton X-100 and humic acids caused a visible decrease in the signal: 2.5 ppm of Triton X—100–80% decrease in the signal; 5 ppm of humic acids—30% decrease in the signal.

### 3.6. Analytical Performance

**Figure 6:**
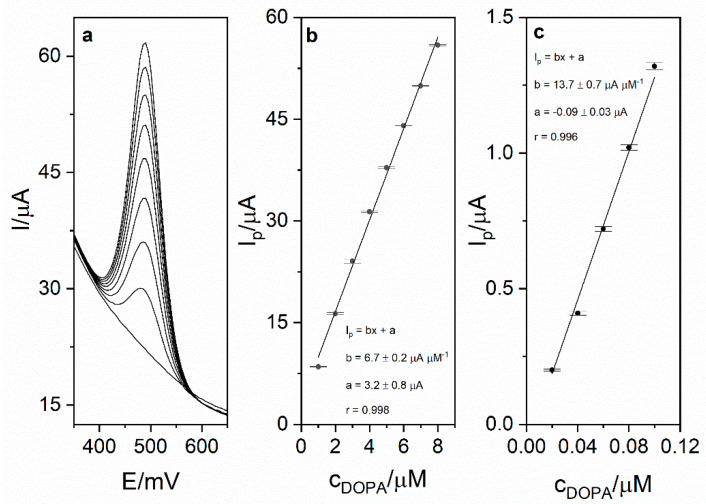
(**a**) DPV calibration voltammogram obtained for levodopa concentrations in the range from 1 to 8 µM and blank in 0.025 M perchloric acid. Preconcentration potential and time were equal to 0 mV and 20 s, respectively. (**b**) Calibration curve for the concentration range 1–8 µM. (**c**) Calibration curve for the range 0.02–0.1 µM.

**Figure 7:**
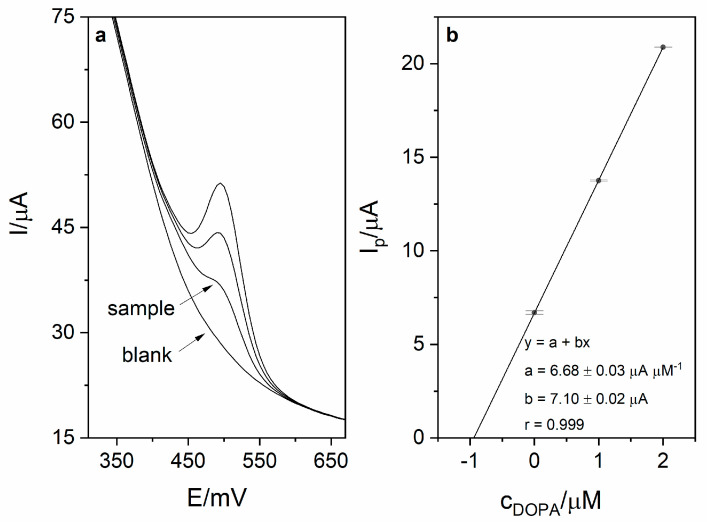
(**a**) DPV voltammogram obtained during sample measurement (Madopar 125) in 0.025 M perchloric acid (blank, sample and 2 additions of levodopa stock solution − 1 µM each). Preconcentration potential and time were equal to 0 mV and 20 s, respectively. (**b**) Corresponding linear dependence.

Calibration was carried out in order to investigate the analytical performance of the voltammetric method for levodopa determination. Calibration voltammograms as well as calibration curves are presented in Figure 6. The linearity was obtained for the levodopa concentration range 1–8 µM (r = 0.998; slope 6.74 ± 0.16 µA µM^−1^, intercept 3.16 ± 0.83 µA). Based on the obtained results (calibration presented in Figure 5), the limit of detection LOD (defined as 3 standard deviation of blank) was calculated and was equal to 17 nM (20 s preconcentration time). The limit of quantification (LOQ) was also calculated and was equal to 56 nM. Obtained sensitivity of the method was very high (6.74 µA µM^−1^). The comparison of the analytical performance of the developed method with other works concerning levodopa are presented in Table 2. As it might be seen, the developed method is characterized by the second best sensitivity and third best LOD value among the works presented in the table. It is also worth mentioning that the sensor used in this work is easy to prepare, which is a major advantage over other sensors described in the literature and presented in Table 2. The repeatability of the method for levodopa concentration 1 µM (*n* = 5) was calculated and was equal to 2.1%. In order to verify the applicability of the developed method, measurements of levodopa concentration in pharmaceuticals products were carried out (exemplary voltammogram presented in Figure 7a). For this purpose, two pharmaceutical products were chosen—Madopar 62.5 (50 mg of levodopa per tablet) and Madopar 125 (100 mg of levodopa per tablet). Measurements were performed in accordance with the procedure described in point 2.5 and the standard addition method. Results presented in Table 3 are in a good agreement with the producer’s declaration. Calculated recoveries were in the range 102–105%, which confirms sufficient accuracy of the method. The analysis of levodopa concentration in the tap water sample was also performed. The obtained results prove that the developed method might be used in the monitoring of drug pollution in tap water samples.

## 4. Conclusions

A new, highly sensitive Adsorptive Stripping Voltammetric method for levodopa determination was developed. An electrochemical sensor based on a GC electrode modified with carbon black, RuO_2_·xH_2_O and Nafion was used. This modification allowed an improvement in sensitivity of up to 12 times compared to the bare GC electrode (the sensitivity of the sensor 6.74 µA µM^−1^). The best results were achieved in the supporting electrolyte consisting of 0.025 M perchloric acid (pH 1.4) with a preconcentration time and potential equal to 20 s and 0 mV, respectively. Limit of detection (LOD) was calculated and was equal to 17 nM. This is a very good result in comparison with solutions previously described in the literature (Table 1), especially because preparation of the presented sensor is extremely simple and fast. Calculated recoveries proved the accuracy of the method. The applicability of the developed method was confirmed by the analysis of levodopa concentration in pharmaceutical products and tap water. The results of drug analysis were in a good agreement with the producer’s declaration. Considering the presented facts, it might be concluded that the developed method might be a useful tool for the quality control of pharmaceutical products and monitoring of drug pollution in the environment.

## Figures and Tables

**Table 1:** Interfering agents used in the experiment.

Interfering Agent	Concentration in the Solution
Fe(III), Se(IV), Cd(II), Pb(II), Zn(II), Al(III), Sb(III), Cu(II), Mn(II)	1 µM
Mg(II), Ca(II)	10 µM
SO_4_^2−^, H_2_PO_4_^−^	1000 µM
citric acid	1000 µM
ascorbic acid, glucose	500 µM
Triton X-100	2.5 ppm
humic acids	5 ppm

**Table 2:** Comparison of previously published results concern levodopa determination.

Electrode	LOD, nM	Sensitivity, µA µM^−^^1^	Reference
3D GF/ITO	1000	0.24	[18]
DyNW/CPE	4	17.46	[19]
GCE MoPD/MWNTs	101	0.80	[21]
EA CPE	650	0.06	[22]
GCE C_60_-MWNTs	35	0.01	[23]
RTIL-GQDs/CPE	10	0.87	[24]
CAMCPE	650	0.17	[25]
ZnS NPs/3D GF	43	2.57	[26]
CB-RuO_2_-Nafion GC	17	6.74	This work

3D GF/ITO—3D graphene foam/ indium tin oxide, DyNW/CPE—dysprosium nanowire carbon paste electrode, GCE moPD/MWNTs—glassy carbon electrode modified with poly 4-methyl-ortho-phenylenediamine and multi-wall carbon nanotubes, EA CPE—electrochemically activated carbon paste electrode, GCE C_60_-MWCNTs—glassy carbon electrode modified with fullerene-functionalized multi wall carbon nanotubes, RTIL-GQDs/CPE—a room temperature ionic liquid/graphene quantum dots modified carbon paste electrode, CAMCPE—chloranil modified carbon paste electrode, ZnS NPs/3D GF—ZnS nanoparticles modified three-dimensional graphene foam.

**Table 3:** Results of levodopa determination in pharmaceutical products and in tap water.

DOPA Added	DOPA Found ± s (Recovery, %), mg		DOPA Found ± s (Recovery, %), µg
	Madopar 62.5 ^a^	Madopar 125 ^b^	Tap Water
0	51 ± 2	102 ± 3	ND
2 µg	-	-	2.1 ± 0.1 (105)
4 µg	-	-	4.1 ± 0.1 (102)
50 mg	104 ± 2 (103)	-	-
100 mg	-	205 ± 7 (102)	-

^a^ 50 mg per tablet declared, ^b^ 100 mg per tablet declared, ND—not detected.
